# Biohybrid Nanocellulose–Lysozyme Amyloid Aerogels via Electrostatic Complexation

**DOI:** 10.1021/acsomega.1c05069

**Published:** 2021-12-23

**Authors:** Leonardo Severini, Kevin J. De France, Deeptanshu Sivaraman, Nico Kummer, Gustav Nyström

**Affiliations:** † Department of Chemical Sciences and Technologies, University of Rome “Tor Vergata”, Via della Ricerca Scientifica 1, 00133 Rome, Italy; ‡ Laboratory for Cellulose & Wood Materials, Empa—Swiss Federal Laboratories for Materials Science and Technology, Überlandstrasse 129, 8600 Dübendorf, Switzerland; § Laboratory for Building Energy Materials and Components, Empa, Swiss Federal Laboratories for Materials Science and Technology, Überlandstrasse 129, CH-8600 Dübendorf, Switzerland; ∥ Department of Health Science and Technology, ETH Zürich, Schmelzbergstrasse 9, 8092 Zürich, Switzerland

## Abstract

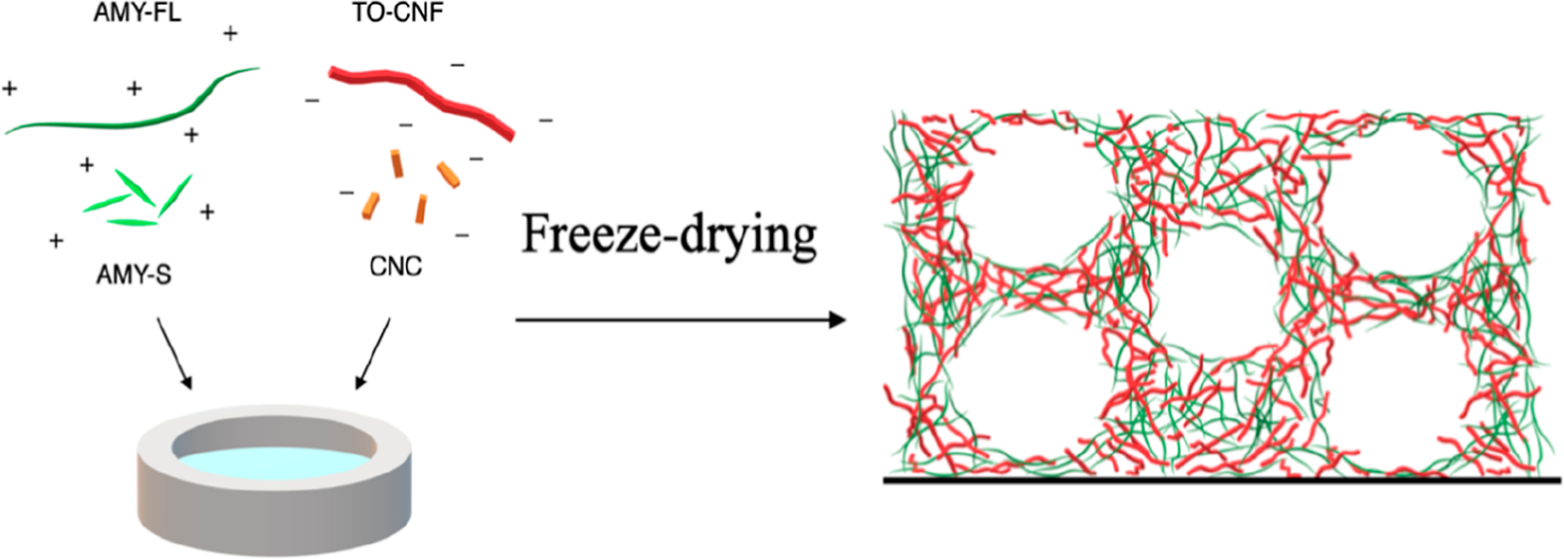

Modern science is increasingly turning to nature for inspiration to design sustainable biomaterials in a smart and effective way. Herein, we describe biohybrid aerogels based on electrostatic complexation between cellulose and proteins—two of the most abundant natural polymers on Earth. The effects of both particle surface charge and particle size are investigated with respect to aerogel properties including the morphology, surface area, stability, and mechanical strength. Specifically, negatively charged nanocellulose (cellulose nanocrystals and cellulose nanofibers) and positively charged lysozyme amyloid fibers (full-length and shortened via sonication) are investigated in the preparation of fibrillar aerogels, whereby the nanocellulose component was found to have the largest effect on the resulting aerogel properties. Although electrostatic interactions between these two classes of charged nanoparticles allow us to avoid the use of any cross-linking agents, the resulting aerogels demonstrate a simple additive performance as compared to their respective single-component aerogels. This lack of synergy indicates that although electrostatic complexation certainly leads to the formation of local aggregates, these interactions alone may not be strong enough to synergistically improve bulk aerogel properties. Nevertheless, the results reported herein represent a critical step toward a broader understanding of biohybrid materials based on cellulose and proteins.

## 1 Introduction

Through evolution and adaptation, natural biological materials perform a variety of specific roles with high efficiency in mild conditions.^1^ As a result, there is growing interest in taking inspiration directly from nature in order to develop sustainable and highly functional biobased materials. Due to their biocompatibility, biodegradability, and a range of chemical functionalities, these biobased materials have attracted significant attention in a wide range of fields such as biomedical, renewable energy, cultural heritage preservation, and building technology, representing a sustainable alternative to petroleum-based resources.^2−10^ Cellulose and proteins are two of the most abundant biopolymers on Earth, playing diversified roles in many biological systems^11−14^ and, as a result, are commonly used as natural building blocks in the preparation of biobased materials such as hydrogels and aerogels. In particular, due to their light weight, high specific surface area, and porous structure, aerogels commonly find application as oil/chemical absorbents, thermal insulators, biomedical scaffolds, and biocatalysts; however, before reaching practical application, the fundamental properties and interactions within biobased aerogels need to be thoroughly investigated.^15^

Nanocelluloses are a class of fibril-like nanoparticles, comprising packed cellulose polymer chains with at least one dimension in the nanoscale.^16^ In general, nanocelluloses exhibit good strength, stiffness, and easily tunable surface chemistry,^17^ making them ideal candidates for the fabrication of structured materials, including aerogels, finding application in areas such as insulating materials, biomedical scaffolds, and adsorbents.^18−23^ Depending on the preparation method employed, nanocelluloses can be divided into cellulose nanofibers (CNFs, long and semiflexible fibers with both highly regular crystalline and less well-ordered domains, typically produced via mechanical fibrillation) and cellulose nanocrystals (CNCs, shorter and more rigid fibers with a high degree of crystallinity, typically produced via acid hydrolysis).^17,24^ Due to the increased flexibility and high aspect ratio of CNFs, they tend to form physically entangled gel networks at low concentrations, while due to the increased rigidity and crystallinity of CNCs, they are often used as mechanical reinforcing agents in composite materials.^17,25^

Protein amyloid fibrils are a class of elongated ordered supramolecular filaments, stabilized by β-sheet secondary structures, and formed via the self-assembly of protein/peptide fragments.^26−28^ Moreover, due to their flexible structure, amyloid fibers can also form entangled networks at relatively low concentrations, which is ideal for the formation of aerogels.^29,30^ In general, protein amyloids are extremely versatile, due to the functional diversity in the protein/peptide moieties they are formed from; this has led to their application in areas such as catalysis, filtration, drug delivery, and biomimetic functional materials.^31,32^ To this end, lysozyme has gained particular interest for its ability to rapidly form amyloid aggregates with broad spectrum antibacterial resistance.^33−36^ This is of particular interest as lysozyme in its native form demonstrates little to no antibacterial efficacy against Gram-negative bacteria.^37^

Despite the great potential of nanocellulose and protein amyloids, their combination to form composite materials has not been widely investigated. Notably, the Freire group prepared composite films from CNFs and lysozyme amyloids, demonstrating the potential of such materials for both water purification and wound healing applications.^38,39^ Here, films were formed via vacuum filtration of the mixed two-component suspensions, whereby the presence of lysozyme amyloid fibers led to an increase in heavy metal removal from contaminated water and increases in antioxidant and antimicrobial activity (against Gram-positive *Staphylococcus aureus*) versus CNF-only films. In all cases, the film’s mechanical properties were relatively similar, albeit with decreased elongation at break (increased brittleness) apparent upon lysozyme amyloid addition.^38,39^ Similarly, work in our group previously investigated the preparation of structured composite films from CNCs and lysozyme amyloids via an evaporation-induced self-assembly.^40^ Although the electrostatic complexation between negatively charged CNCs and positively charged lysozyme amyloids led to films with a decreased structural order, the incorporation of lysozyme amyloids improved the film’s antimicrobial properties against Gram-positive *S. aureus*, again with relatively small effects on film mechanics. Taken together, these results suggest that the incorporation of protein amyloids into nanocellulose-based materials is an effective method to add functionality based on the amyloid component, with the nanocellulose component providing structural integrity. To this end, herein, we prepare electrostatically complexed biohybrid aerogels from nanocellulose (CNC and TO-CNF) and lysozyme amyloids [full-length (AMY-FL) and sonicated (AMY-S)]. The effects of nanocellulose and lysozyme morphology are investigated with respect to aerogel properties including the porosity, stability, and mechanical strength.

## 2 Results and Discussion

**Figure 1:**
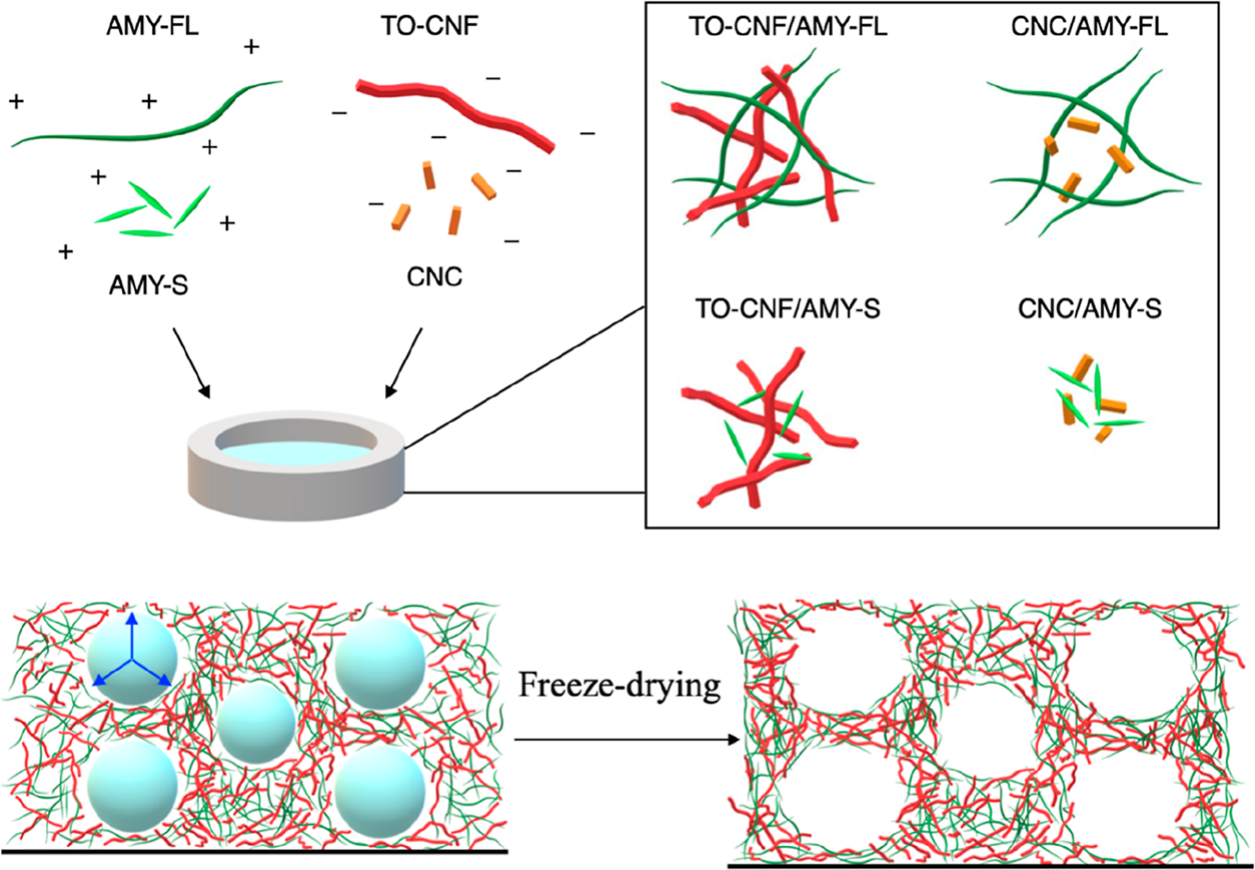
Schematic representation of nanocellulose (TO-CNF or CNC) and lysozyme amyloid (AMY-FL or AMY-S) components used as building blocks in the preparation of biohybrid aerogels. Biohybrid mixtures were frozen using liquid nitrogen and freeze-dried in order to obtain the corresponding aerogels.

**Figure 2:**
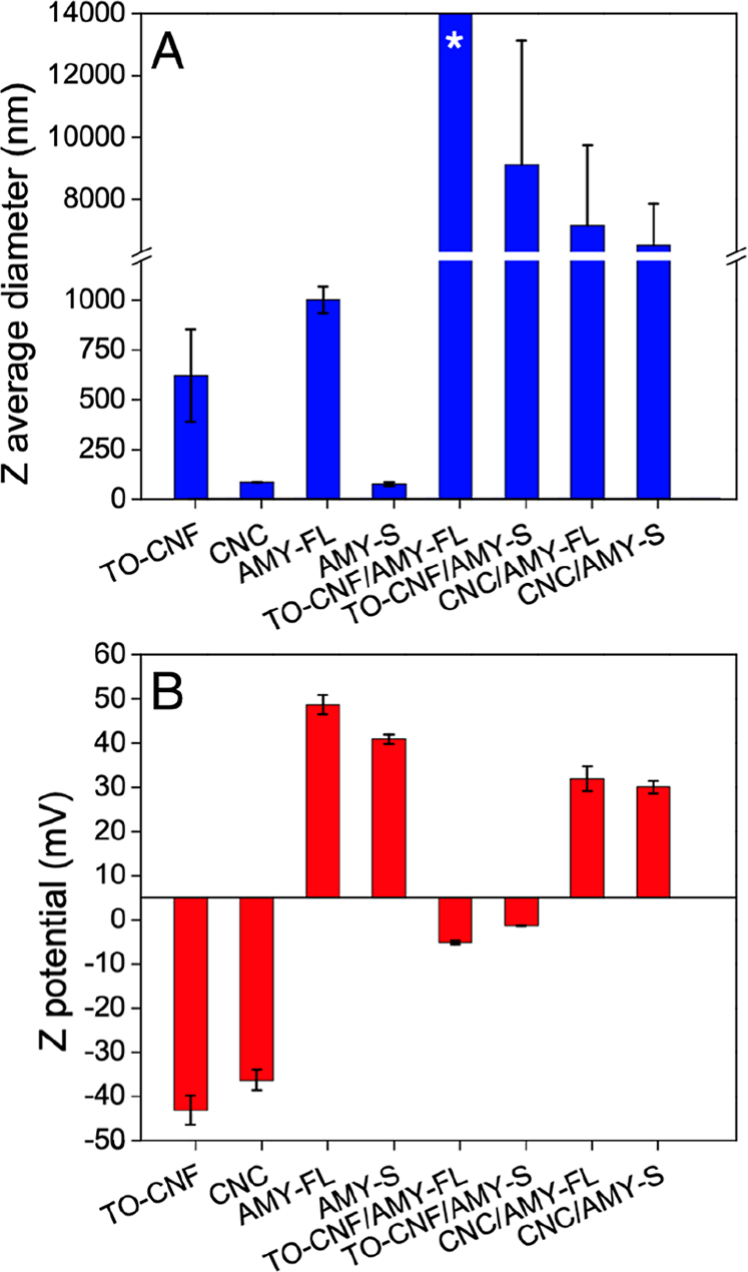
Suspension characterizations for the single-component and biohybrid mixtures: (A) *z*-average diameter and (B) zeta potential values. * Apparent size larger than the maximum measurable value for the DLS instrument.

Nanocellulose–lysozyme amyloid biohybrid suspensions were formed via simple electrostatic complexation between negatively charged nanocellulose (CNC zeta potential = −36.3 ± 2.3 mV and TO-CNF zeta potential = −43.1 ± 3.3 mV) and positively charged lysozyme amyloid fibrils (AMY-FL zeta potential = 48.7 ± 2.2 mV and AMY-S zeta potential = 40.9 ± 1.1 mV). This process is shown schematically in Figure 1. Vortex mixing 2 wt % suspensions at a 1:1 ratio resulted in the immediate formation of colloidal aggregates (optical photographs, Supporting Information, Figure S2), indicated by the increase in suspension turbidity. Aggregation was also apparent via dynamic light scattering (DLS), with *z*-average diameters in the micron range (Figure 2). The aggregate size followed the trend of the nanoparticle size, whereby TO-CNF/AMY-FL suspensions showed the largest *z*-average diameter (apparent size larger than 10 μm) and CNC/AMY-S suspensions showed the smallest (6.5 ± 1.3 μm). This suggests that nanoparticle entanglement plays a large role in the aggregate network formation via electrostatic complexation. These biohybrid networks were visualized via scanning electron microscopy (SEM) (Supporting Information, Figure S3), whereby, in general, suspensions containing TO-CNFs appeared to have a more compact pore structure with larger pores and suspensions containing CNCs appeared to have a more open pore structure with smaller pores. No clear differences were evidenced between samples containing AMY-FL and AMY-S, this suggests that the nanocellulose component has a greater impact on the network morphology than the lysozyme amyloid component. As nanocellulose is commonly incorporated into composite materials as a key structural component,^21,41^ here it also shows the greatest effect on the porosity/pore structure.

**Figure 3:**
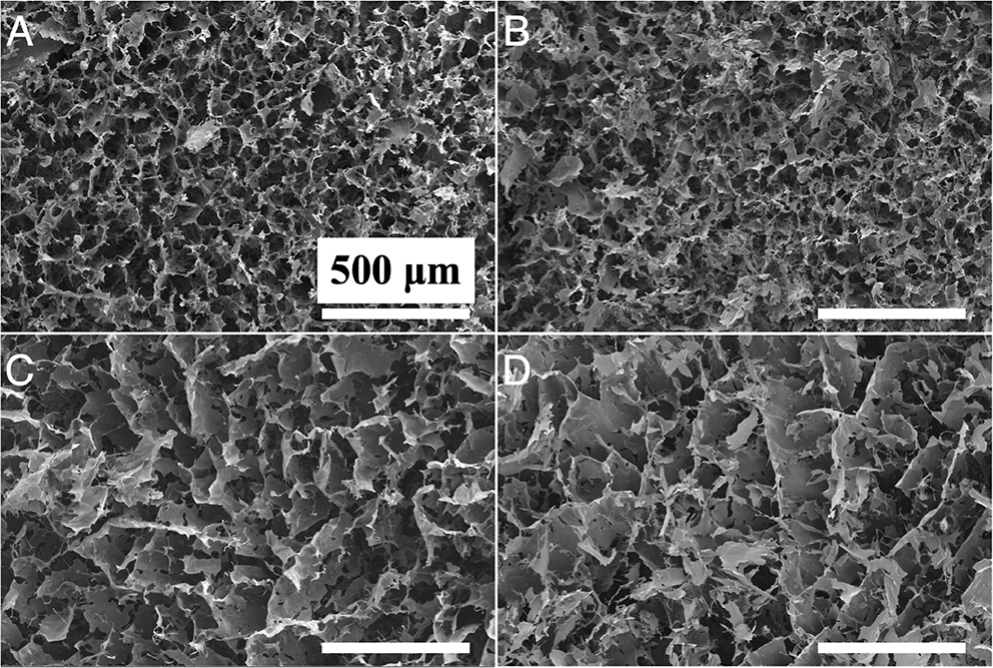
SEM images of biohybrid aerogels: (A) TO-CNF/AMY-FL, (B) TO-CNF/AMY-S, (C) CNC/AMY-FL, and (D) CNC/AMY-S. All scale bars are 500 μm.

Aerogels were formed via simple freeze-drying of the single-component and biohybrid suspensions described above. Suspensions were directly submerged in liquid nitrogen, whereby ice crystal growth compresses the particles/biohybrid aggregates, forcing increased interactions via hydrogen bonding, hydrophobic interactions, and physical entanglement. In general, aerogels containing TO-CNF were relatively compact and easy to handle, while aerogels containing CNC were fluffier and more delicate (single-component and biohybrid aerogels can be seen in Supporting Information, Figure S4). This can be attributed to the biohybrid aggregate morphology observed via SEM, as discussed above. All formed aerogels demonstrated a relatively low degree of shrinkage (calculated as the final aerogel cross-sectional area divided by the cross-sectional area of the mold used, Supporting Information, Table S1), indicating the maintenance of an intact internal porous structure. Interestingly, TO-CNF/AMY-FL aerogels showed the highest degree of shrinkage (13.5 ± 0.3%), while CNC/AMY-S aerogels showed the lowest (7.2 ± 0.1%), following the trend for the aggregate size discussed above. This is intuitive as the relatively flexible nature of TO-CNF and AMY-FL would allow for increased compression/compaction during freezing as compared to the relatively rigid and less entangled CNC and AMY-S. Note that the poorer shape fidelity of the CNC-containing aerogels likely also leads to an over-estimation of the cross-sectional area due to the fluffy nature of these samples. Correspondingly, a biohybrid aerogel morphology was assessed via SEM (Figure 3), whereby aerogels containing TO-CNF exhibited a more compact pore structure than aerogels containing CNC. SEM images of the single-component aerogels can be seen in the Supporting Information (Figure S5).

In all cases, aerogels exhibited a relatively low specific surface area, as expected due to the freeze-drying process used (Supporting Information, Table S2).^22,25^ Because the process for the gel matrix and aerogel production involve freezing and immobilization of the amyloid fibers and CNFs with electrostatic forces and not chemical/physical bonding, we expect to see larger pores (see Figure 3 for SEM images) in comparison to other freeze-dried aerogels and supercritically dried aerogels.^42,43^ This kind of pore structure and processing traditionally results in lower Brunauer–Emmett–Teller (BET) surface areas, as they are outside the mesoporous range (2–50 nm). Here, TO-CNF-containing aerogels showed the highest BET specific surface area (∼20 m^2^/g) as compared to CNC-containing aerogels (∼3 m^2^/g). This is most likely attributed to the pore collapse of the relatively fluffy CNC-containing aerogels during both the freeze-drying process and aerogel handling/BET sample preparation. Here again, relatively minimal effects of the amyloid component were evidenced on aerogel porosity, supporting the hypothesis of the nanocellulose component acting as the key structural building block in these biohybrid composites. Sorption isotherms for all aerogel samples can be seen in the Supporting Information (Figure S6), whereby a type IV H2/H1 isotherm with large macropores but some mesoporosity dominates for CNF-containing aerogels, while an isotherm more of type II with large macropores and almost no mesopores dominates for CNC-containing aerogels.^44,45^

**Figure 4:**
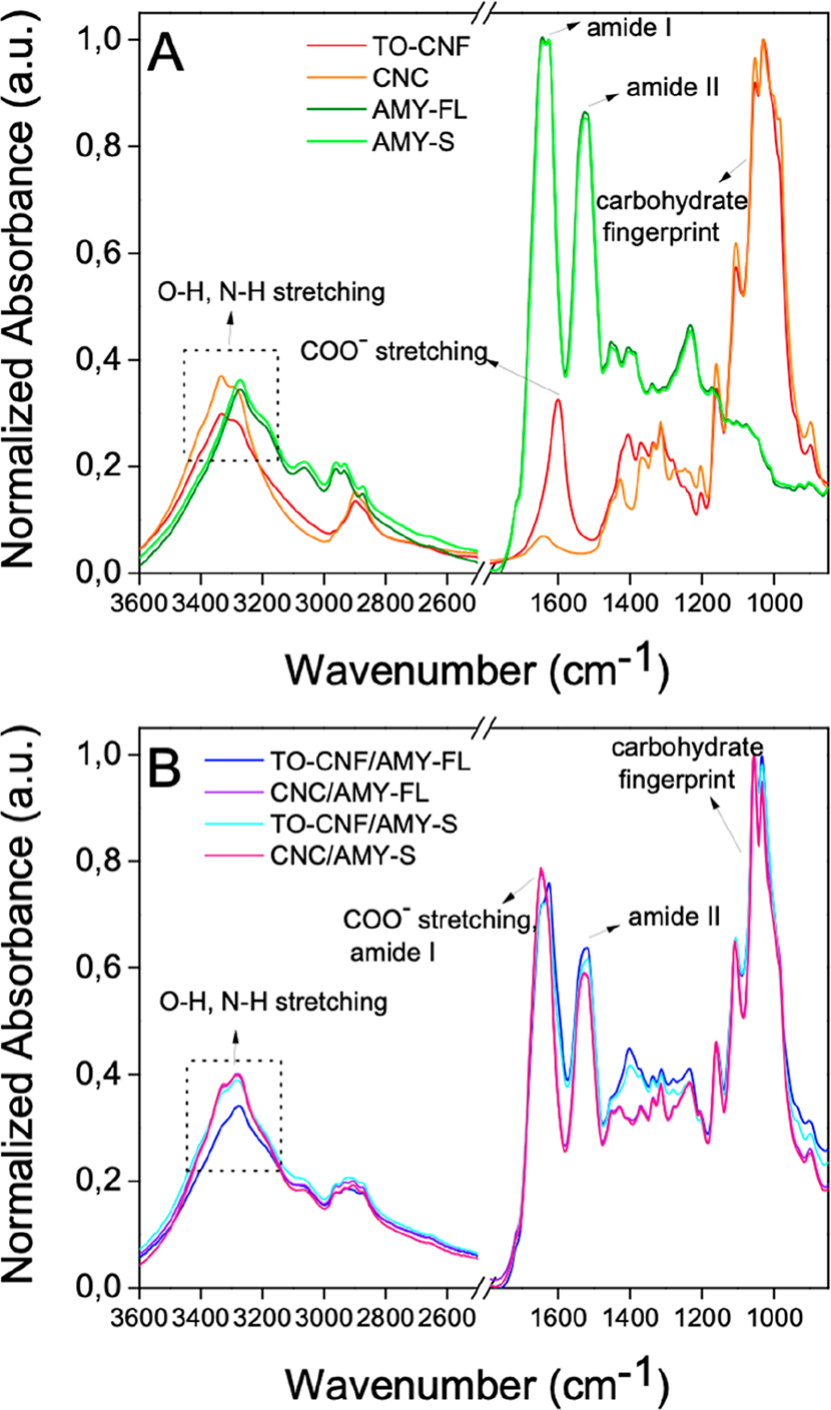
Normalized absorbance ATR–FTIR spectra in the range of 3600–800 cm^–1^: (A) single-component and (B) biohybrid aerogels. Several characteristic peaks are highlighted.

The aerogel chemical composition was assessed via Fourier transform infrared (FTIR) (Figure 4); pure nanocellulose aerogels displayed broad peaks at ∼3340 cm^–1^ and ∼2900/∼1430 cm^–1^, which are attributed to O–H stretching vibrations and C–H stretching/bending vibrations, respectively. A characteristic carbohydrate fingerprint is also apparent between 900 and 1280 cm^–1^.^46,47^ Additionally, TO-CNF aerogels displayed a peak at ∼1600 cm^–1^, related to the presence of carboxylate moieties from the 2,2,6,6-tetramethyl-1-piperidinyloxyl (TEMPO) oxidation process. Pure lysozyme amyloid aerogels also displayed broad peaks at ∼3340 cm^–1^ due to O–H and N–H stretching vibrations^48,49^ but in addition showed strong vibrational bands attributed to amide I (∼1650 cm^–1^) and amide II (∼1540 cm^–1^) modes, which are very sensitive to protein secondary structure/conformational changes. Here, the amide I band is mainly attributed to C=O stretching vibrations belonging to backbone polypeptide chains; the amide II band arises from a combination of N–H in-plane bending, C–N stretching, and other amide group vibrations.^50−53^ The deconvolution of AMY-FL and AMY-S aerogel spectra was performed in order to evaluate the effects of sonication on the protein secondary structure (Supporting Information, Table S3).^54^ In both cases, a high content of the β-sheet structure, as expected for protein amyloids,^40,55−57^ was evidenced, with almost no variation before and after sonication. This suggests that sonication is an effective method for tuning amyloid fibril length without affecting its protein conformational state. Expectedly, biohybrid aerogels displayed peak characteristic for both nanocellulose and lysozyme amyloids, indicating the successful incorporation of both components.

Aerogel stability was assessed both thermogravimetrically (Supporting Information, Figure S7 and Table S4) and in water (Supporting Information, Figure S8). In all cases, thermogravimetric profiles (Figure S7A) follow a classic two-stage trend for biological materials; in the first stage (generally below 200 °C) relatively minimal mass-loss is evidenced, corresponding mainly to the evaporation of absorbed and intermolecular H-bonded water. In the second stage (generally above 200 °C), depolymerization and degradation of biopolymer chains are prevalent and result in considerable mass loss.^57^ The onset of thermal degradation values (Table S4) shows that CNC aerogels are the most thermally stable (287 °C), while TO-CNF aerogels are the least stable, with the onset of thermal degradation beginning at 224 °C. This difference in thermal stability can be attributed to the surface charge density and degree of crystallinity; previous studies have shown that nanocellulose functionalized with sulfate groups exhibits greater thermal stability than nanocellulose functionalized with carboxylic groups. Moreover, previous studies have also demonstrated that nanocellulose with a higher degree of crystallinity (which is the case for CNC vs TO-CNF) shows a higher onset of thermal degradation.^58−60^ The onset of thermal degradation for both AMY-FL and AMY-S is intermediate to that of TO-CNF and CNC, but importantly both AMY-FL and AMY-S exhibit an identical degradation behavior, indicating that the sonication process does not affect the thermal stability of the resulting amyloid fibers. Expectedly, biohybrid aerogels exhibited intermediate thermal degradation profiles compared to their single-component counterparts. Notably, only aerogels containing TO-CNF showed any stability in water; this suggests that TO-CNF network entanglement is crucial, and electrostatic complexation alone cannot guarantee aerogel stability. Intuitively, AMY-FL fibrils are also long and flexible, and therefore should be able to form entangled network structures similar to TO-CNF, however little stability in water was observed for these aerogels. This likely has to do with the overall conversion of native lysozyme into its amyloid state; previous research has demonstrated that mature lysozyme amyloid fibers still contain a significant portion of the non-structured lysozyme,^36^ as the entire peptide sequence does not become involved in amyloid fiber formation.^55,61^ Therefore here, the presence of the native/non-structured lysozyme likely hinders network formation as there is a lower concentration of the structured fibers within the aerogels.

**Figure 5:**
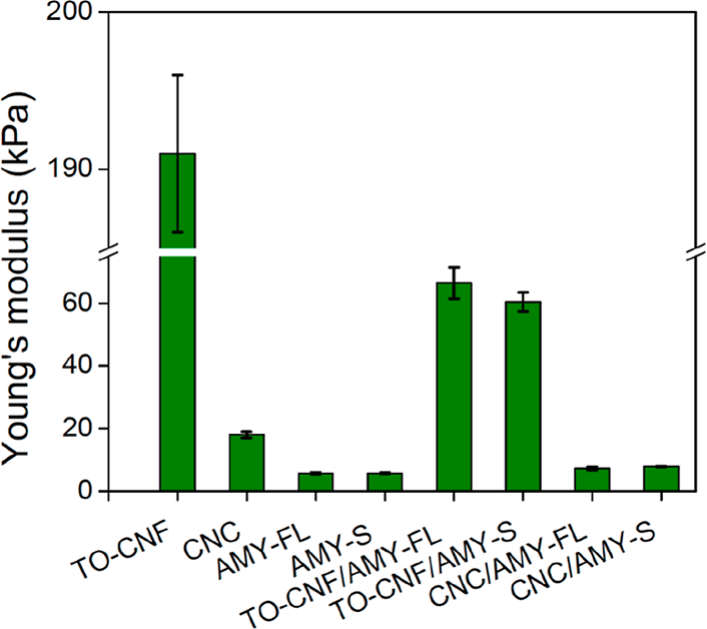
Young’s modulus values for aerogel samples as determined via compression. The results are presented as the average with error bars representing the standard deviation, whereby at least four samples were measured for each aerogel. Representative stress–strain curves are presented in the Supporting Information (Figure S9A).

In order to study and compare aerogel mechanical performance, compressive stress–strain testing was performed (Supporting Information, Figure S9), whereby Young’s modulus values were calculated from the initial linear viscoelastic region (Figure 5). For single-component aerogels, Young’s modulus values for TO-CNF aerogels (191 ± 5 kPa) were an order of magnitude larger than for any of the other single-component aerogels, again demonstrating the importance of network entanglement. Conversely, aerogels deriving from rigid CNCs show a lamellar internal structure, which generally yields poor mechanical properties.^62^ Similarly for AMY-FL and AMY-S, the resulting aerogels are relatively weak and fragile due to an internal plate-like morphology. Biohybrid aerogels showed intermediate Young’s modulus values between their respective single-component counterparts, demonstrating simple additive performance. This lack of synergistic reinforcement via electrostatic complexation perhaps indicates that although the formation of local aggregation occurs between negatively charged nanocellulose and positively charged lysozyme amyloids, this does not necessarily lead to a globally complexed structure. To further test this hypothesis, biohybrid aerogels containing a 10:1 ratio of nanocellulose to lysozyme amyloids (a ratio that we have previously shown to be synergistic in other materials)^40^ were prepared, and Young’s modulus values were determined (Supporting Information, Figure S10). Here again, a simple additive performance between the two network components was observed with no evidence of synergistic enhancements in the network strength. This likely indicates that internal regions containing a higher concentration of lysozyme amyloids act as weak points within the gel network, which are more susceptible to compression. Note that in all cases, the aerogels demonstrate poor shape recovery, suggesting that additional (chemical) cross-linking may be required for applications where cyclic loading and unloading cycles are foreseen.

**Figure 6:**
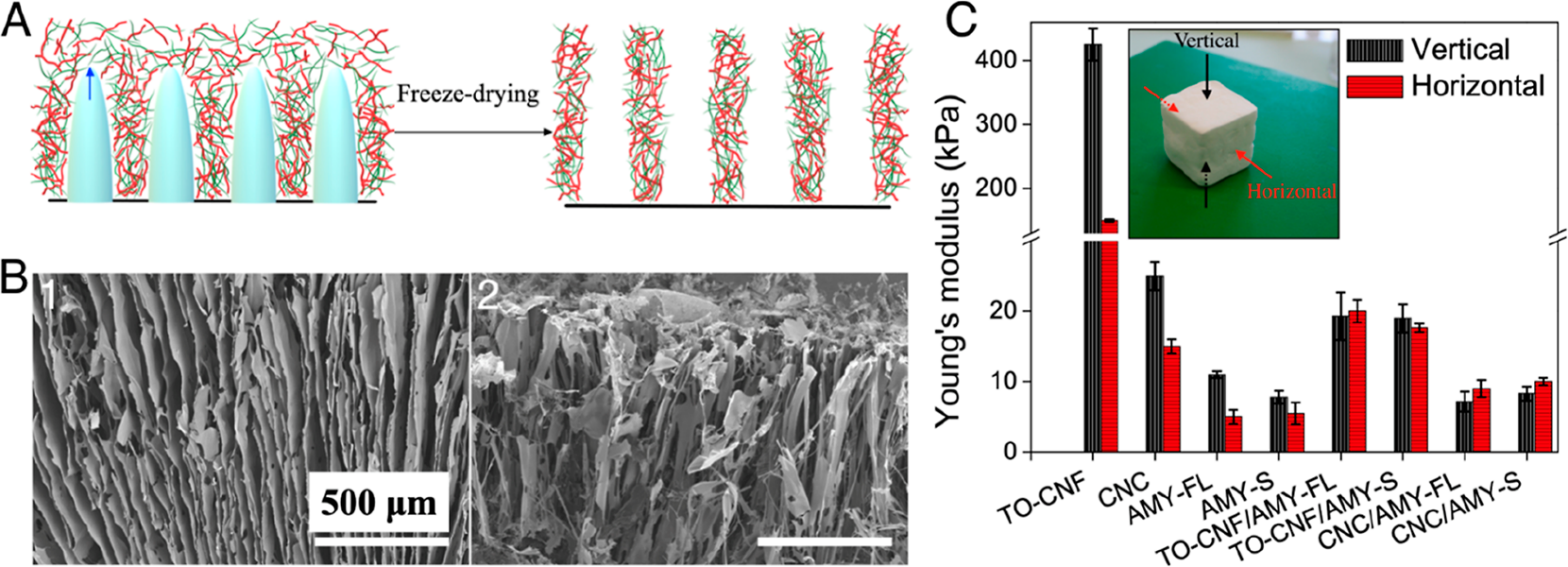
(A) Schematic illustration of the ice-templating process to prepare single-component and biohybrid aerogels via liquid nitrogen-induced directional freezing. (B) SEM images of directionally frozen aerogels: (1) CNC and (2) CNC/AMY-FL respectively. (C) Young’s modulus values in the vertical (parallel to the freezing direction) and horizontal (perpendicular to the freezing direction) directions for ice-templated aerogel samples. The results are presented as the average with error bars representing the standard deviation, whereby at least four samples were measured for each condition.

Finally, the effects of the freezing process were investigated on aerogel formation via the preparation of directionally frozen single-component and biohybrid aerogels (Figure 6). Directional freezing—or ice-templating—is a process whereby a solidifying solvent (water) progresses uniaxially through a dispersed phase, which is excluded to form an organized and anisotropic pore wall structure after sublimation.^63,64^ Here, SEM images of single-component aerogels sectioned parallel to the ice crystal growth direction reveal a typical and relatively homogeneous aligned pore morphology, consistent with other biobased aerogels prepared via similar techniques (CNC shown in Figure 6, all others in the Supporting Information, Figure S11).^65,66^ For all single-component aerogels, as seen in Figure 6, this enhanced pore orientation leads to enhanced mechanical reinforcement along this axis (vertical) with respect to the perpendicular one (horizontal). This is due to the aligned/lamellar pore structure providing increased resistance in the direction of applied stress. Interestingly, directionally frozen biohybrid aerogels do not exhibit a regular aligned pore morphology (SEM image for CNC/AMY-FL shown in Figure 6, all others in Supporting Information, Figure S11); this is likely due to the formation of local electrostatically complexed aggregates hindering ice-crystal growth. It is well known that the formation of a regular anisotropic pore morphology depends heavily on the effective rejection of particles in the dispersed phase by the advancing ice front.^63,64^ As the particle size increases, the ability to effectively exclude these particles and form a homogeneous pore morphology decreases;^63^ here, we hypothesize that the locally formed aggregates likely act as larger particles themselves, disrupting the freezing process and leading to a more heterogeneous pore morphology. Correspondingly, these biohybrid aerogels do not exhibit any prominent mechanical anisotropy, with Young’s modulus values similar in both horizontal and vertical directions (Figure 6). Taken together, these results suggest that locally, electrostatic complexation can be a prominent contributor to aggregate formation; however, across larger length scales, these interactions are insufficient to produce robust biohybrid aerogels and can even hinder bulk material properties.^67^

## 3 Conclusions

In conclusion, biohybrid aerogels were successfully prepared via electrostatic complexation between negatively charged nanocellulose (TO-CNFs and CNCs) and positively charged lysozyme amyloid fibrils (AMY-FL and AMY-S). In general, the nanocellulose component appeared to play the biggest role in a biohybrid aerogel morphology and mechanical properties, with CNF-based aerogels having a more compact and organized pore structure and CNC-based aerogels showing a more open and plate-like pore morphology. Additionally, the mechanical properties were much better for the CNF-based biohybrid aerogels than the CNC-based biohybrid aerogels; however, in both cases, these aerogels demonstrated a simple additive performance as compared to their respective single-component aerogel counterparts. This indicates that although electrostatic complexation was evidenced between the nanocellulose component and the lysozyme amyloid component, these interactions alone are not strong enough to result in any bulk synergistic effects. This is supported by SEM images of directionally frozen aerogels, whereby single-component gels exhibit a typical anisotropic pore morphology and mechanical properties, whereas biohybrid aerogels exhibit a much more disorganized structure and relatively isotropic mechanical properties, attributed to local electrostatic aggregates hindering ice crystal growth during the freezing process. Taken together, the results presented here represent a meaningful step toward a broader understanding of biohybrid aerogels based on nanocellulose and protein amyloid fibrils and the interactions therein.

## 4 Experimental Section

### 4.1 Materials

CNCs (prepared via sulfuric acid hydrolysis of bleached softwood kraft pulp) were purchased in a freeze-dried form from CelluForce (Montreal, Canada). CNCs were probe-sonicated (10 min, 60% amplitude, Digital Sonifier 450, Branson Ultrasonics) at a concentration of 2 wt % in distilled water and refrigerated until use. Never-dried elemental chlorine-free cellulose fibers from bleached softwood pulp (*Picea abies* and *Pinus* app.) were obtained from Stendal GmbH (Berlin, Germany) and used for the preparation of TEMPO-oxidized CNFs (TO-CNFs) following our previously published protocol.^67^ The prepared TO-CNFs were concentrated to 2 wt % and refrigerated until further use. Hen egg white lysozyme (HEWL, >90%), (3-aminopropyl)triethoxysilane (APTES), and sodium hydroxide (NaOH) were purchased from Sigma-Aldrich. TEMPO, sodium hypochlorite (NaClO) solution (12–14% chlorine), hydrochloric acid (HCl), and sodium chloride were purchased from VWR. Sodium bromide (NaBr ≥99%) was supplied by Carl Roth GmbH & Co. All chemicals were used as received.

### 4.2 Preparation of Lysozyme Amyloid Fibrils (Full Length and Sonicated)

Lysozyme amyloid fibrils were prepared via our previously published protocol.^40^ Briefly, the HEWL powder was dissolved in a Falcon tube at a concentration of 2 wt % in distilled water, and the pH was adjusted to 2.0 using 1.0 M HCl. The HEWL solution was then placed in a thermomixer (Eppendorf AG) set to 90 °C and mixed at 400 rpm for 24 h to produce full-length amyloid fibrils (AMY-FL).^40^ Optionally, AMY-FL were then sonicated (10 min, 2 s pulse, 30% amplitude) to generate shorter fibers (AMY-S).^36^ The lysozyme amyloid fibril suspensions were stored at 4 °C in a fridge until use.

### 4.3 Preparation of Single-Component and Biohybrid Aerogels

Aerogels were prepared from single-component and mixed (1:1 by weight) biohybrid suspensions, having a total solid content of 2 wt % in all cases. For the single-component aerogels, 2 wt % suspensions were directly frozen in liquid nitrogen and stored in a −80 °C freezer overnight prior to freeze-drying (FreeZone 4.5, Labconco, USA). For the biohybrid aerogels, HEWL amyloid fibrils (AMY-FL or AMY-S) were vortex-mixed for 1 min with equal volumes of nanocellulose (TO-CNFs or CNCs) to obtain four different biohybrid combinations. These mixtures were then directly frozen and freeze-dried following the same procedure as for single-component aerogels. This process is shown schematically, along with aerogel nomenclature, in Figure 1. As a comparison, directional freezing was also performed for all cases, whereby suspensions were poured into Teflon molds and placed on a stainless-steel plate partially submerged in liquid nitrogen to induce gradient ice crystal growth. As before, samples were then placed in a −80 °C freezer overnight prior to freeze-drying. Prepared aerogel samples were stored at room temperature prior to use/characterization.

### 4.4 Characterization

Single-component and biohybrid suspensions were characterized via DLS (0.025 wt % in water) and zeta potential measurements (0.25 wt % in 10 mM NaCl) using a ZetaSizer Nano ZS (Malvern). AFM measurements (Supporting Information, Figure S1, Bruker ICON3) were performed in the tapping mode (0.05 mg/mL in pH 2 Milli-Q for AMY-FL and AMY-S on mica substrates; 0.005 mg/mL in Milli-Q for TO-CNFs and CNCs on 0.05% APTES-coated mica substrates). Single-component and biohybrid aerogels were characterized via FTIR spectroscopy in the attenuated total reflectance (ATR) mode (Bruker Switzerland AG). Spectra were recorded between 4000 and 400 cm^–1^ with a resolution of 4 cm^–1^ and 32 scans per sample. Deconvolution was performed in the 1360–1800 cm^–1^ region on normalized absorbance ATR-FTIR spectra by means of Voigt bands of full width at half height (FWHH) of 25 cm^–1^ maximum, using OMNIC software. Aerogel cross sections and internal porosity were assessed via SEM (FEI NanoSEM 230). Samples were coated with a thin layer of platinum (∼2 nm) before imaging (5.0 mm working distance and 5.0 kV accelerating voltage). Compression testing was performed using a Z010 device (Zwick) equipped with a 20 N load cell. A compression rate corresponding to ∼10% of the initial specimen height per min was used for all measurements. The samples were compressed to 80% strain, whereby Young’s modulus was calculated from the initial linear portion of the stress–strain curves. Compression testing was repeated in at least quadruplicate, with results presented as the average ± standard deviation. Thermal properties of aerogels were analyzed using a Netzsch TG 209F1 thermogravimetric analyzer operating at 10 K min^–1^. Nitrogen adsorption–desorption isotherms were carried out on a Micromeritics 3 Flex (Micromeritics, USA) device, and the surface areas were calculated by the BET method. A sample of the measured mass (30–40 mg) was placed in a special glass sample tube and degassed to 0.016 mbar for 15 h at 85 °C prior to the measurements. The samples were weighed again, and the nitrogen sorption isotherms were analyzed at 77 K from *P*/*P*_0_ ranging from 0.001 to 0.998 in 50 steps, with a maximum equilibration time of 600 s for each incremental nitrogen addition, for a total run time of ∼6 h.

## Supporting Information Available

AFM images of single-component suspensions, photographs of 2 wt % single-component suspensions and 1:1 biohybrid mixtures, SEM images of combination mixtures, additional SEM images of isotropically frozen single-component aerogels and directionally frozen aerogels, photographs of single-component aerogels, aerogel’s shrinkage values, peak assignment, mean values and FWHH used for amyloid spectral deconvolution, thermogravimetric and temperature/derivative analysis of aerogels, onset of thermal degradation, maximum degradation rate and temperature at the maximum degradation rate of aerogels, stress–strain curves of aerogels, photographs of 2 wt % single-component and biohybrid aerogels in Milli-Q water, isotropic Young’s modulus values of 10:1 biohybrid aerogels, and BET measurements of aerogels (PDF)
