# Supplementary material for: Biohybrid Nanocellulose–Lysozyme Amyloid Aerogels via Electrostatic Complexation
Source: ACS Omega. 2021 Dec 23;7(1):578–86. doi: 10.1021/acsomega.1c05069 (PMC8757363; doi:10.1021/acsomega.1c05069)
Supplement: Supplementary file 1 — ao1c05069_si_001.pdf [file ao1c05069_si_001.pdf]

Supporting Information

Biohybrid Nanocellulose – Lysozyme Amyloid Aerogels via Electrostatic Complexation

Leonardo Severini [a, b], Kevin J. De France [b], Deeptanshu Sivaraman [c], Nico Kummer [b, d], and Gustav Nyström * [b, d]

corresponding author email: gustav.nystroem@empa.ch

[a] Department of Chemical Sciences and Technologies, University of Rome "Tor Vergata", Via della Ricerca Scientifica 1, 00133 Rome, Italy

[b] Laboratory for Cellulose & Wood Materials, Empa–Swiss Federal Laboratories for Materials Science and Technology, Überlandstrasse 129, 8600 Dübendorf, Switzerland

[c] Laboratory for Building Energy Materials and Components, Empa, Swiss Federal Laboratories for Materials Science and Technology, Überlandstrasse 129, Dübendorf CH-8600, Switzerland

[d] Department of Health Science and Technology, ETH Zürich, Schmelzbergstrasse 9, 8092 Zürich, Switzerland

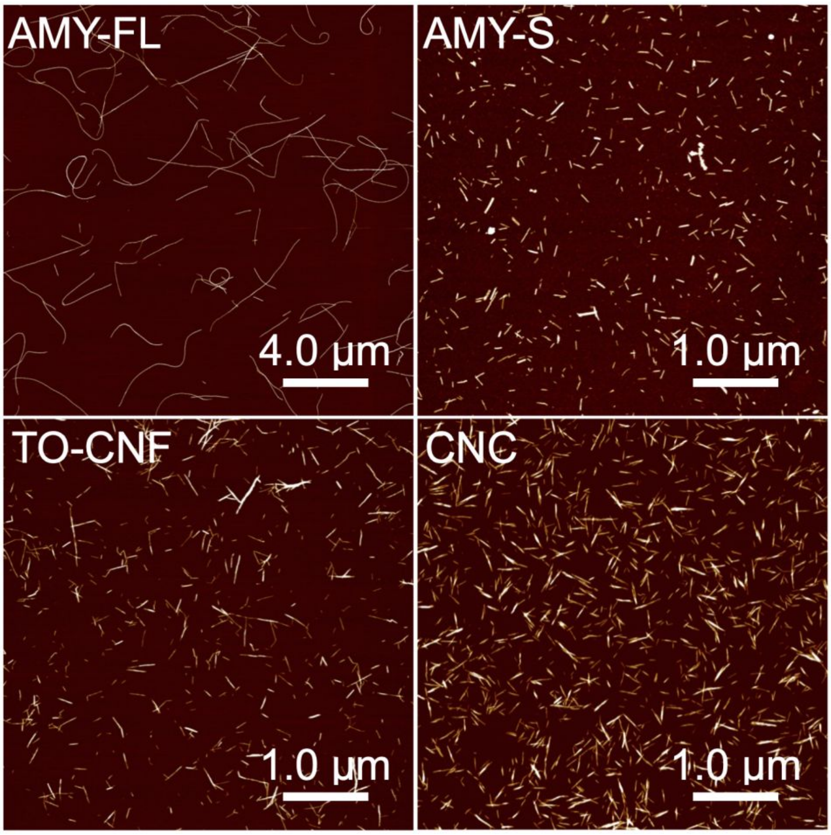

Figure S1. AFM images of single-component suspensions.

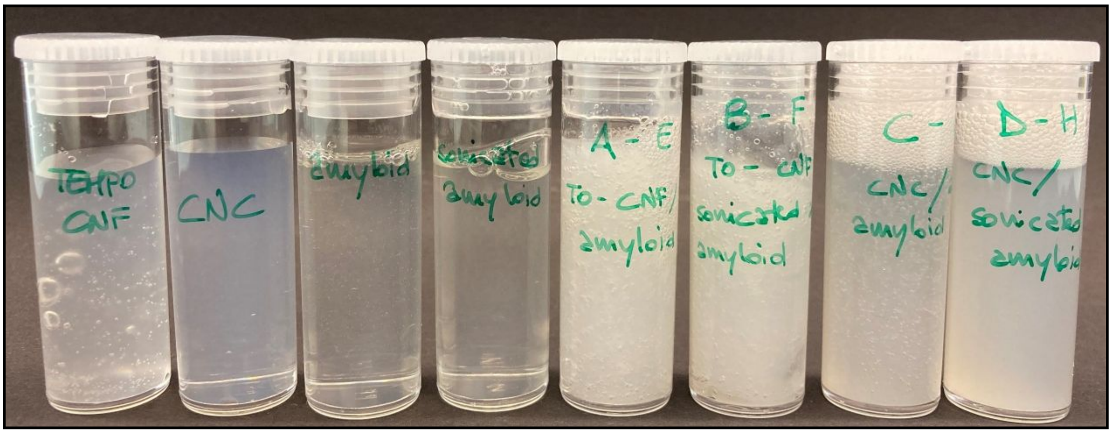

Figure S2. Photographs of 2 wt % single-component suspensions and 1:1 biohybrid mixtures. From left: TO-CNF, CNC, AMY-FL, AMY-S, TO-CNF/AMY-FL, TO-CNF/AMY-S, CNC/AMF-FL, CNC/AMY-S. The increased

turbidity of the 1:1 biohybrid mixtures is indicative of local aggregation between the negatively charged nanocellulose component and the positively charged lysozyme amyloid component.

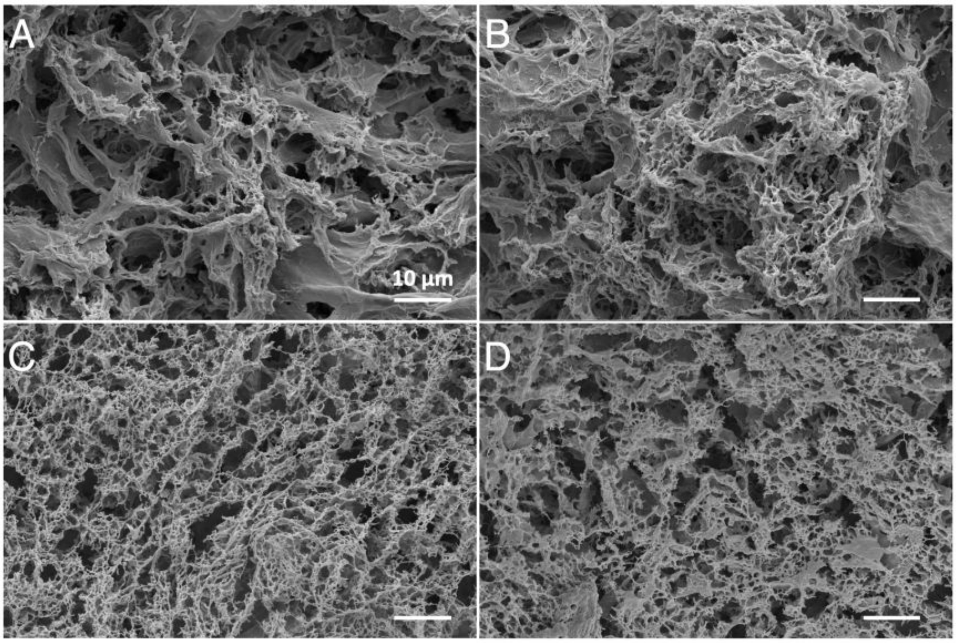

Figure S3. Scanning electron microscopy images of 2 wt% biohybrid mixtures. Here, suspensions were drop-cast onto SEM sample holders prior to coating with a thin layer of platinum before imaging. Respectively: (A) TO-CNF/AMY-FL, (B) TO-CNF/AMY-S (C) CNC/AMF-FL and (D) CNC/AMY-S. All scale bars are 10 µm.

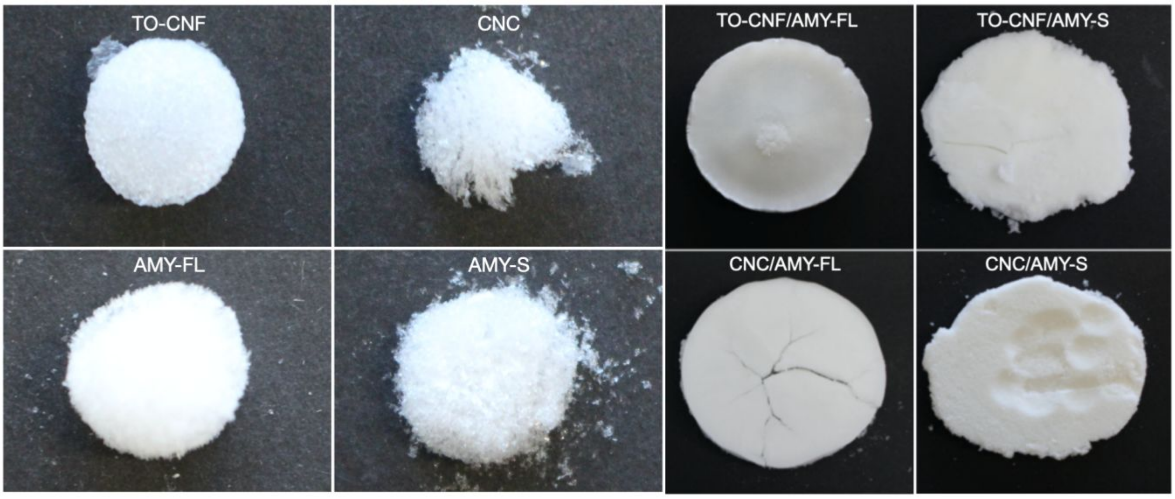

Figure S4. Photographs of single-component and biohybrid aerogels.

Table S1. Measured aerogel shrinkage following freeze drying. Shrinkage is calculated based on the cross-sectional area of the prepared aerogels compared to the cross-sectional area of the tubes they were prepared in.

**Sample:**	**Isotropically forzen aerogel shrinkage (%)**	**Directionally frozen aerogel shrinkage (%)**
AMY-FL	9.1 ± 0.1	7.2 ± 0.1
AMY-S	9.3 ± 0.2	7.1 ± 0.3
TO-CNF	9.0 ± 0.1	6.4 ± 0.2
CNC	8.4 ± 0.2	6.1 ± 0.5
TO-CNF/AMY-FL	13.5 ± 0.3	11.2 ± 0.1
TO-CNF/AMY-S	10.1 ± 0.2	9.1 ± 0.3
CNC/AMY-FL	8.4 ± 0.2	7.0 ± 0.2
CNC/AMY-S	7.2 ± 0.1	7.0 ± 0.4

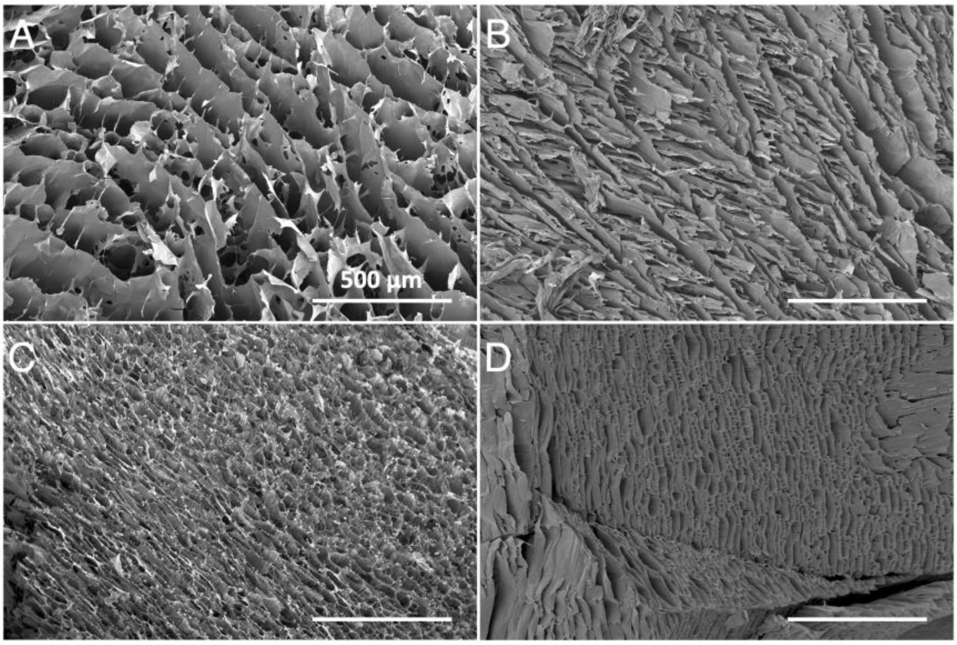

Figure S5. Scanning electron microscopy images of single-component aerogels. Respectively: (A) TO-CNF, (B) CNC, (C) AMY-FL and (D) AMY-S. All scale bars are 500 µm.

Table S2. BET results of aerogel samples including sorption isotherm types, pore size distributions, and specific surface area.

**Sample Name**	**N_2 adsorption–desorption isotherms**	**BJH pore size distributions**	**specific surface area (m^2/g)**
AMY-FL	Type I (b)/Type II	Mostly macroporous	4.8
AMY-S	Type I (b)/Type II	Mostly macroporous	3.3
TO-CNF	Type IV H3	Mostly macroporous, sparse mesoporosity	10
CNC	Type IV H3	Mostly macroporous, sparse mesoporosity	8.1
TO-CNF/AMY-FL	Type IV H3	Mesoporosity 10%<br>Macropores 90%	18
TO-CNF/AMY-S	Type IV H4	Mesoporosity 10%<br>Macropores 90%	23
CNC/AMY-FL	Mostly Type I(b) + Type IV H3	Micropores 5%<br>Mesopores 5%<br>Macropores 90%	3.0

CNC/AMY-S	Mostly Type I(b) + Type IV H3	Micropores 5%<br>Mesopores 5%<br>Macropores 90%	3.5

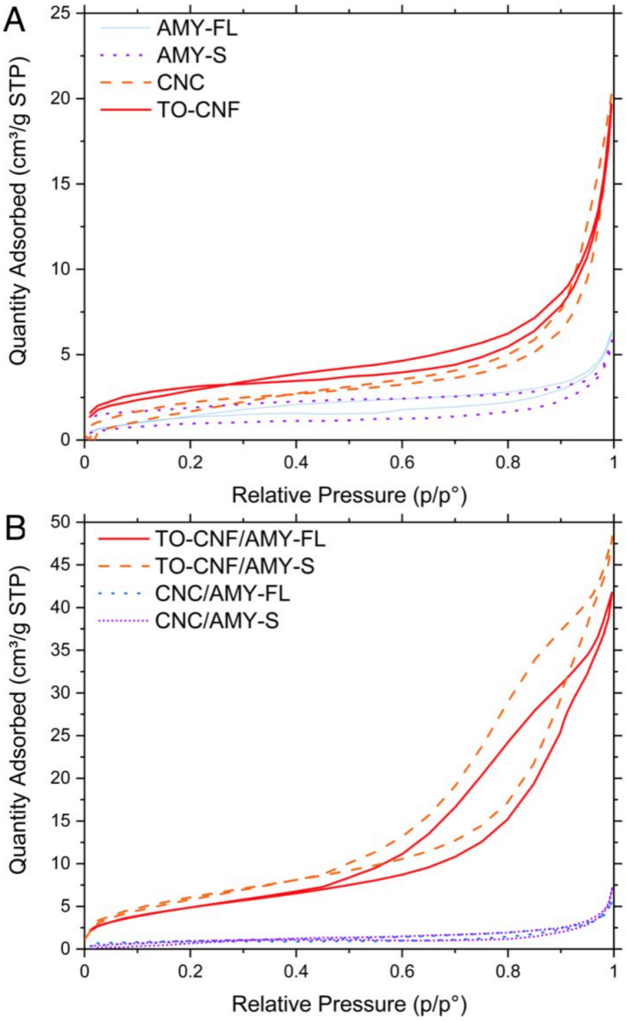

Figure S6. Sorption isotherms obtained from BET analysis on (A) single-component and (B) biohybrid aerogels.

Table S3. Peak assignment, Mean values and FWHH used for FTIR spectra deconvolution

	AMY-FL		AMY-S	
**Peak assigned to:**	**Mean value [a]**	**FWHH [b]**	**Mean value [a]**	**FWHH [b]**
β sheet	1512	42	1511	40
β sheet	1543	37	1543	37
Tyrosine side chain	1594	5	1593	5
β sheet	1622	40	1623	40
Random coil/α helix	1650	32	1650	30
Turns/loops	1672	12	1672	12
β sheet	1690	4	1690	3

[a] Peak values are in a range of 10 cm^{-1}
[b] Values are allowed to vary in a range of 20 cm^{-1}

Table S4. Thermogravimetric analysis showing onset of thermal degradation, max degradation rate and Temperature at max degradation rate of aerogel samples

**Aerogel sample**	**Onset of thermal degradation (°C)**	**Max. degradation rate (% mass/°C)**	**Temperature at max. degradation rate (°C)**
AMY-FL	260	-4.4/-4	300/337
AMY-S	261	-4.8/-5.8	301/331
TO-CNF	224	-6/-5.6	260/290
CNC	287	-29	304
TO-CNF/AMY-FL	246	3/-6.5	260/325
TO-CNF/AMY-S	245	3/-7	260/325
CNC/AMY-FL	270	-7.5	340
CNC/AMY-S	265	-6.2	332

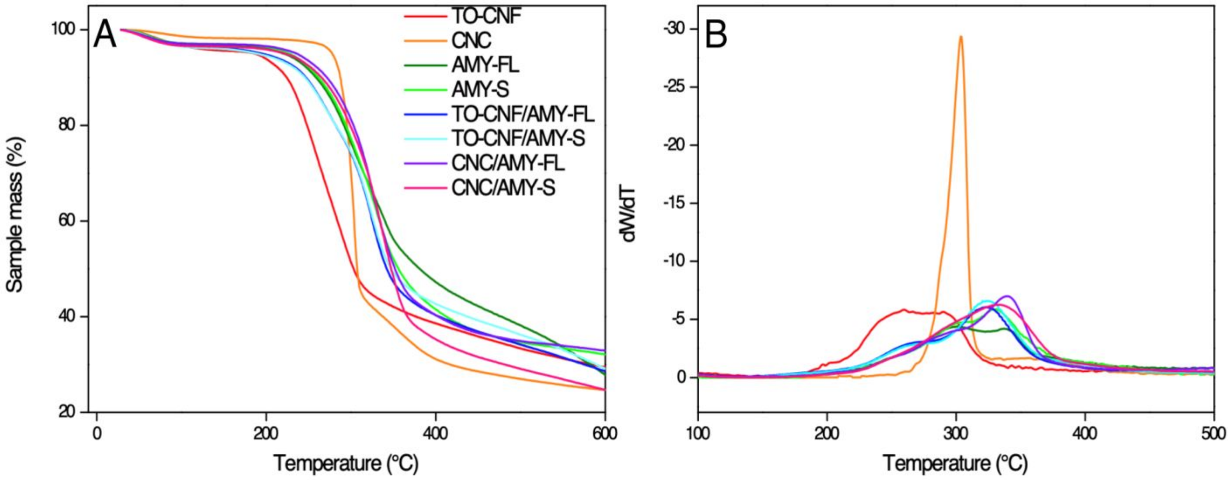

Figure S7. (A) Thermogravimetric and (B) temperature/derivative analysis of aerogels.

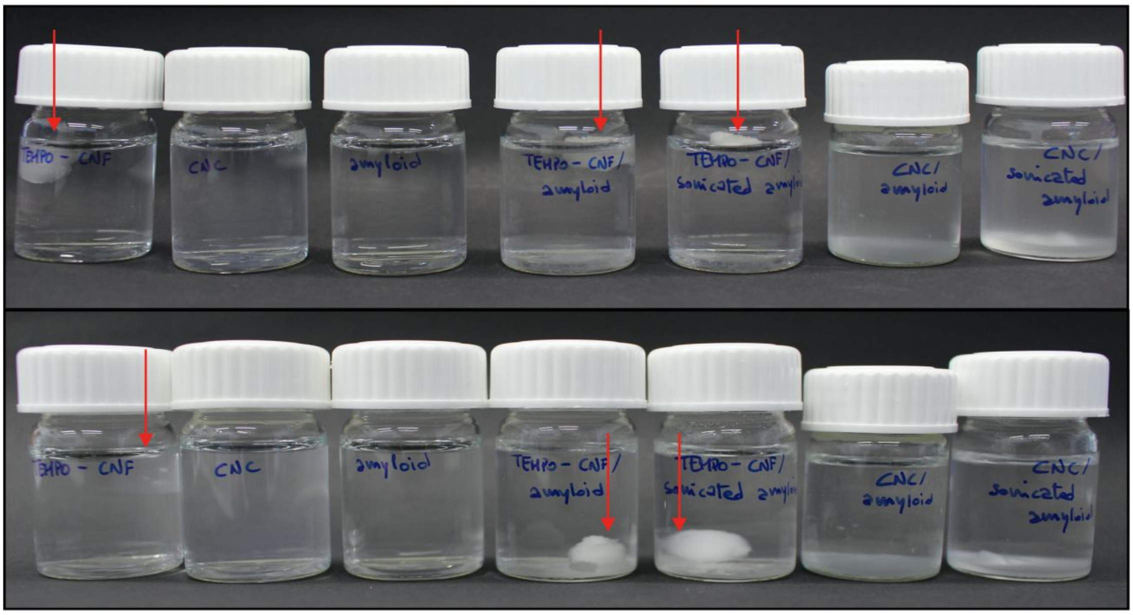

Figure S8. Photographs of 2 wt % single-component and biohybrid aerogels in Milli-Q water. Upper panel: day 1; lower panel: day 30. Arrows indicate the aerogels which demonstrate some shape fidelity after submersion in water.

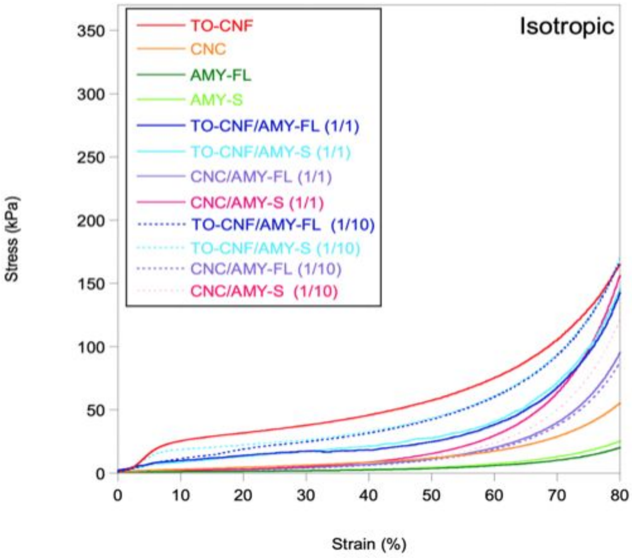
Isotropic
TO-CNF
CNC
AMY-FL
AMY-S
TO-CNF/AMY-FL (1/1)
TO-CNF/AMY-S (1/1)
CNC/AMY-FL (1/1)
CNC/AMY-S (1/1)
TO-CNF/AMY-FL (1/10)
TO-CNF/AMY-S (1/10)
CNC/AMY-FL (1/10)
CNC/AMY-S (1/10)
Stress (kPa)
Strain (%)

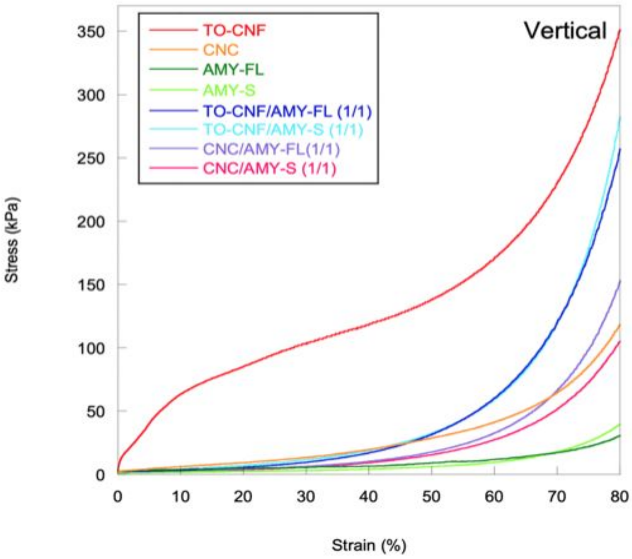
Vertical
TO-CNF
CNC
AMY-FL
AMY-S
TO-CNF/AMY-FL (1/1)
TO-CNF/AMY-S (1/1)
CNC/AMY-FL(1/1)
CNC/AMY-S (1/1)
Stress (kPa)
Strain (%)

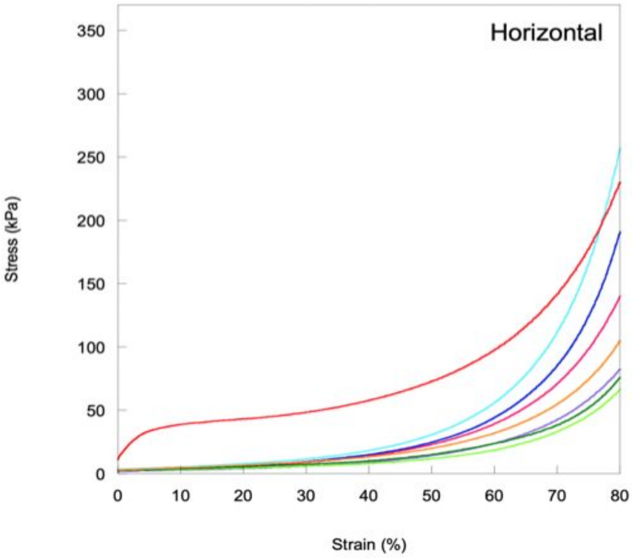
Horizontal
Stress (kPa)
Strain (%)

Figure S9. Representative compressive stress–strain curves for 2 wt % aerogels tested in: isotropic, vertical and horizontal directions, respectively.

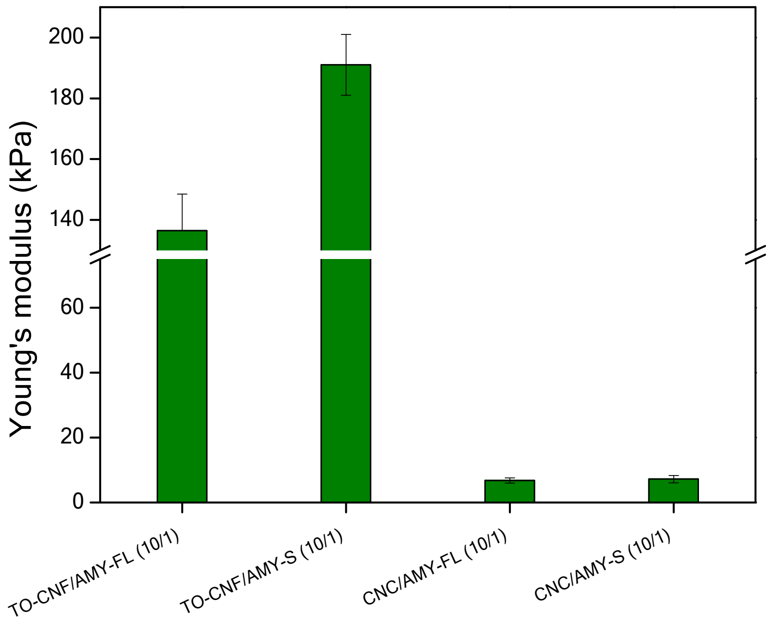

Figure S10. Young’s moduli values of biohybrid aerogels containing a 10:1 ratio of nanocellulose to amyloids prepared via isotropic freezing.

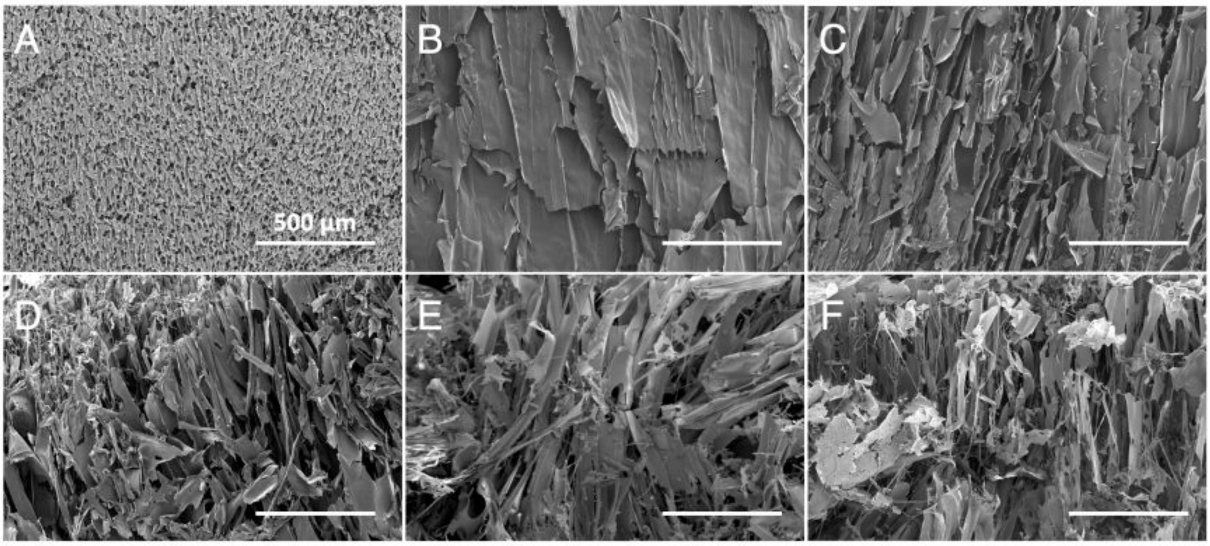

Figure S11. Scanning electron microscopy images of directionally frozen aerogels. Respectively: (A) TO-CNF, (B) AMY-FL, (C) AMY-S, (D) TO-CNF/AMY-FL, (E) TO-CNF/AMY-S, (F) CNC/AMY-S. All scale bars are 500 μm.
